# Baseline prepulse inhibition dependency of orexin A and REM sleep deprivation

**DOI:** 10.1007/s00213-024-06555-3

**Published:** 2024-03-01

**Authors:** Pınar Öz, Osman Kamalı, Hacer Begüm Saka, Ceren Gör, İsmail Tayfun Uzbay

**Affiliations:** 1 Department of Molecular Biology and Genetics, Üsküdar University, Istanbul, Turkey; 2 Faculty of Engineering and Natural Sciences, Üsküdar University Central Campus Block A, Altunizade Mah. Haluk Türksoy Sk. No : 14 34362, Üsküdar, Istanbul Turkey; 3 Department of Neuroscience, Üsküdar University, Istanbul, Turkey; 4 Department of Neuroscience, Koç University, Istanbul, Turkey; 5 Faculty of Medicine, Üsküdar University, Istanbul, Turkey

**Keywords:** Orexin A, Prepulse inhibition, REM sleep deprivation, Psychosis

## Abstract

**Rationale:**

Prepulse inhibition (PPI) impairment reflects sensorimotor gating problems, i.e. in schizophrenia. This study aims to enlighten the role of orexinergic regulation on PPI in a psychosis-like model.

**Objectives:**

In order to understand the impact of orexinergic innervation on PPI and how it is modulated by age and baseline PPI (bPPI), chronic orexin A (OXA) injections was carried on non-sleep-deprived and sleep-deprived rats that are grouped by their bPPI.

**Methods:**

bPPI measurements were carried on male Wistar rats on P45 or P90 followed by grouping into low-PPI and high-PPI rats. The rats were injected with OXA twice per day for four consecutive days starting on P49 or P94, while the control groups received saline injections. 72 h REMSD was carried on via modified multiple platform technique on P94 and either OXA or saline was injected during REMSD. PPI tests were carried out 30 min. after the last injection.

**Results:**

Our previous study with acute OXA injection after REMSD without bPPI grouping revealed that low OXA doses might improve REMSD-induced PPI impairment. Our current results present three important conclusions: (1) The effect of OXA on PPI is bPPI-dependent and age-dependent. (2) The effect of REMSD is bPPI-dependent. (3) The effect of OXA on PPI after REMSD also depends on bPPI.

**Conclusion:**

Orexinergic regulation of PPI response with and without REMSD can be predicted by bPPI levels. Our findings provide potential insights into the regulation of sensorimotor gating by sleep/wakefulness systems and present potential therapeutic targets for the disorders, where PPI is disturbed.

**Supplementary Information:**

The online version contains supplementary material available at 10.1007/s00213-024-06555-3.

## Introduction

Recent studies suggest an intriguing involvement of the orexinergic system in the etiology of neurodevelopmental and neurodegenerative disorders (Deutch and Bubser 2007; Lambe et al. 2007; Morein-Zamir et al. 2007; Kang et al. 2009; Borgland and Labouebe 2010; Fronczek et al. 2012; Roh et al. 2014). Specifically for schizophrenia, the attention on the sleep-regulated etiology brought forth a possible key role of the orexinergic system. In clinical trials, schizophrenia patients with higher plasma OXA levels had fewer negative and positive symptoms (Chien et al. 2015) and higher plasma level was also correlated with the body-mass index in patients with fewer negative symptoms (Liu et al. 2020). Increased OXA level was suggested as a biomarker in schizophrenia patients, i.e. as a predictor for a link between genetic and social isolation (Ni et al. 2019). Furthermore, antipsychotics, such as clozapine and olanzapine, interact with orexin receptors (Monda et al. 2018; Tiwari et al. 2016) and plasma OXA levels (Başoğlu et al. 2010; Chen et al. 2019). OX1R and OX2R polymorphisms correlate with symptoms such as narcolepsy, extreme daytime drowsiness, polydipsia-hyponatremia in patients with schizophrenia (Meerabux et al. 2005; Fukunaka et al. 2007; Thompson et al. 2014). Additionally, reduced plasma OXA levels were also reported in bipolar disorder patients (Tsuchimine et al. 2019) and in female schizophrenia patients (Lu et al. 2021). These findings indicate that there is indeed an effect of orexin levels on the positive and negative symptoms of schizophrenia and they urge a deeper investigation of the orexinergic system involvement, i.e. via the use of symptomatic animal models. It is also important to note that the orexin expression is reported to alter by age (Aran et al. 2012; Kanbayashi et al. 2002), i.e. the number of orexin-immunoreactive neurons decrease (Hunt et al. 2015) and there is significant loss of orexinergic innervation due to aging (Stanley and Fadel 2012). This decline in orexinergic activity appears to be consistent with the decline in sleep duration and quality with aging.

Prepulse inhibition (PPI) is the inhibition of startle response to a strong sensory stimulus via a preceeding weaker sensory stimulus that does not produce startle response itself (Graham 1975). The impairment in PPI reflects sensorimotor gating dysfunction and can be a diagnostic symptom in various neurodevelopmental and neurodegenerative disorders, such as schizophrenia, autism spectrum disorder, Alzheimer’s Disease and Huntington’s Disease (Swerdlow et al. 1995; Braff et al. 2001; Broberg et al. 2010). PPI impairment is considered a strong and trustable endophenotype especially for schizophrenia (Swerdlow and Light 2016). Since it is evolutionarily conserved, PPI is often used for validation of symptomatic animal models of neurodevelopmental and neurodegenerative disorders (Braff and Geyer 1990; Swerdlow et al. 1995, 2008; Swerdlow and Light 2016). In addition to its utilization as a diagnostic marker and a model validification test, previous studies suggest that low baseline PPI levels of model organisms can be a marker for innate neurodevelopmental problems (Peleg-Raibstein et al. 2013).

Sleep deprivation (SD) produces a similar impact on PPI when compared to neurodevelopmental and neurodegenerative disorders, providing a useful model to focus on the sleep-regulated etiology of these disorders. In humans, experimentally controlled SD led to positive, negative and cognitive symptoms similar to schizophrenia on healthy volunteers including PPI reduction (Petrovsky et al. 2014; Faiola et al. 2018; Meyhöfer et al. 2017, 2019). Therefore, PPI was suggested as a marker to understand psychotic event pathogeny after SD (Petrovsky et al. 2014). Further studies showed that 72 h REMSD similarly reduced PPI in animal models (Frau et al. 2008; Zubedat et al. 2013; Chang et al. 2014). The impairment was reversed by the use of antipsychotics such as haloperidol, clozapine and risperidone, and REMSD was recommended as a partial symptomatic animal model of schizophrenia, due to the similar symptomatic spectrum including the PPI impairment endophenotype (Frau et al. 2008; Kumari and Ettinger 2020).

The relationship between sleep-regulated systems and PPI is yet to be fully revealed, however the orexinergic system can be considered as one of the strongest candidates for this relationship, i.e. due to its involvement in the neurodevelopmental and neurodegenerative disorders that impair PPI and due to the distribution of orexinergic targets on the PPI network (Supp. Fig. 1). The significant PPI impairment in OX1R-knock-out mice in response to 110 dB and 120 dB startle stimuli (Abbas et al. 2015) and reduced PPI response in orexin-deficient mice (Demidova et al. 2022) can be considered as proofs for this modulation.

In a previous study, we addressed this relationship by analyzing the PPI response to a 72 h REMSD and comparing it to acute OXA applications (Öz et al. 2018). Previous studies reported varying impact of SD on the orexinergic activity. The number of GABA-A and GABA-B receptors decrease in the orexinergic neurons (Matsuki et al. 2015; Toossi et al. 2016) and plasma OXA levels also decrease after SD (Ran et al. 2015). As a result, systemic and nasal delivery of OXA was shown to attenuate the effects of SD in nonhuman primates (Deadwyler et al. 2007). On the other hand, during REMSD, nearly the 34% of the orexinergic neurons become activated, leading an increased level of OXA at LC, neocortex and posterior hypothalamus, while the OXA levels in hippocampus and PPTg remain unaltered (Mehta et al. 2015). Yet another study showed that OXA, OX1R and OX2R levels in hippocampus increase after SD and lower doses of OXA increase neuronal viability in hippocampal cultures (Wang et al. 2019). Previous studies also reported increased cerebrospinal fluid orexin levels in correlation with sleep disturbances in schizophrenia (Nishino et al. 2002). These findings pointed towards a yet-to-be-fully-explained variation in the relationship between SD and OXA, therefore, we also tested the impact of acute OXA administration after REMSD. Our findings pointed towards a dose-dependent and opposite effect of OXA on PPI response for REMSD and non-sleep-deprived (NSD) rats. The high acute dose OXA (40 μg/kg) impaired PPI in NSD animals, while the mild acute dose OXA (10 μg/kg) completely restored the impaired PPI% at 78 dB after REMSD, which was the prepulse intensity with the highest PPI% impairment (Öz et al. 2018). These findings raised further questions about the possible role of especially low doses of OXA administration in PPI response and whether there could be a possible therapeutical dose interval. In this study, we targeted three of the follow-up questions:

1. Our previous study did not include grouping over baseline-PPI levels. Here, our first question was the baseline-PPI dependency of response to OXA, i.e. how low chronic doses of OXA would affect low- and high-PPI animals. Furthermore, we previously focused only on the effect of OXA on older animals (P90-100). Therefore, we further expanded our research to include two age groups, P45 and P90, to also investigate the possible age-dependency of the chronic OXA response.
2. Our second question was about the effect of low dose OXA application during REMSD. In this study, this question was only tested on P98 animals, to enable comparison with our previous findings.
3. Our final question was the baseline-PPI-dependency of REMSD response. Since low baseline PPI might already indicate neurodevelopmental problems, it could be expected that REMSD would be ineffective if the underlying systems were overlapping or it could worsen or improve PPI if there was no overlap.

## Material and methods

### Animals

The permission for the experiments was issued by the Üsküdar University Animal Experiments Local Ethics Committee (Ü.Ü-HADYEK 2016–15, date: 23.08.2016). 165 adult male Wistar albino rats at postnatal day 45 (P45) (*n* = 59) and P90 (*n* = 106) were included in the study and 7 rats were excluded with a suspicion of deafness. The animals were maintained at 22 ± 3  C, and relative humidity of %60 ± 5 with a 12 h light/12 h dark cycle (07:00/19:00). The animals were harbored in standard cages (five animals per cage) and they were given ad libitum food and water. The number of subjects in each group is given in Table 1.

### Prepulse inhibition of acoustic startle reflex

PPI% responses were measured using four Acoustic Startle Reflex (ASR) System chambers (SR-LAB, San Diego Instruments, San Diego, CA, USA). Details of this system and the PPI task protocol is given in previous studies (Öz et al. 2018). The average of the background noise was set at 70 dB. Pulse intensity was 120 dB and prepulse intensities (PIs) were 74 dB, 78 dB and 86 dB, preceeding the pulse by 100 ms.

**Fig. 1:**
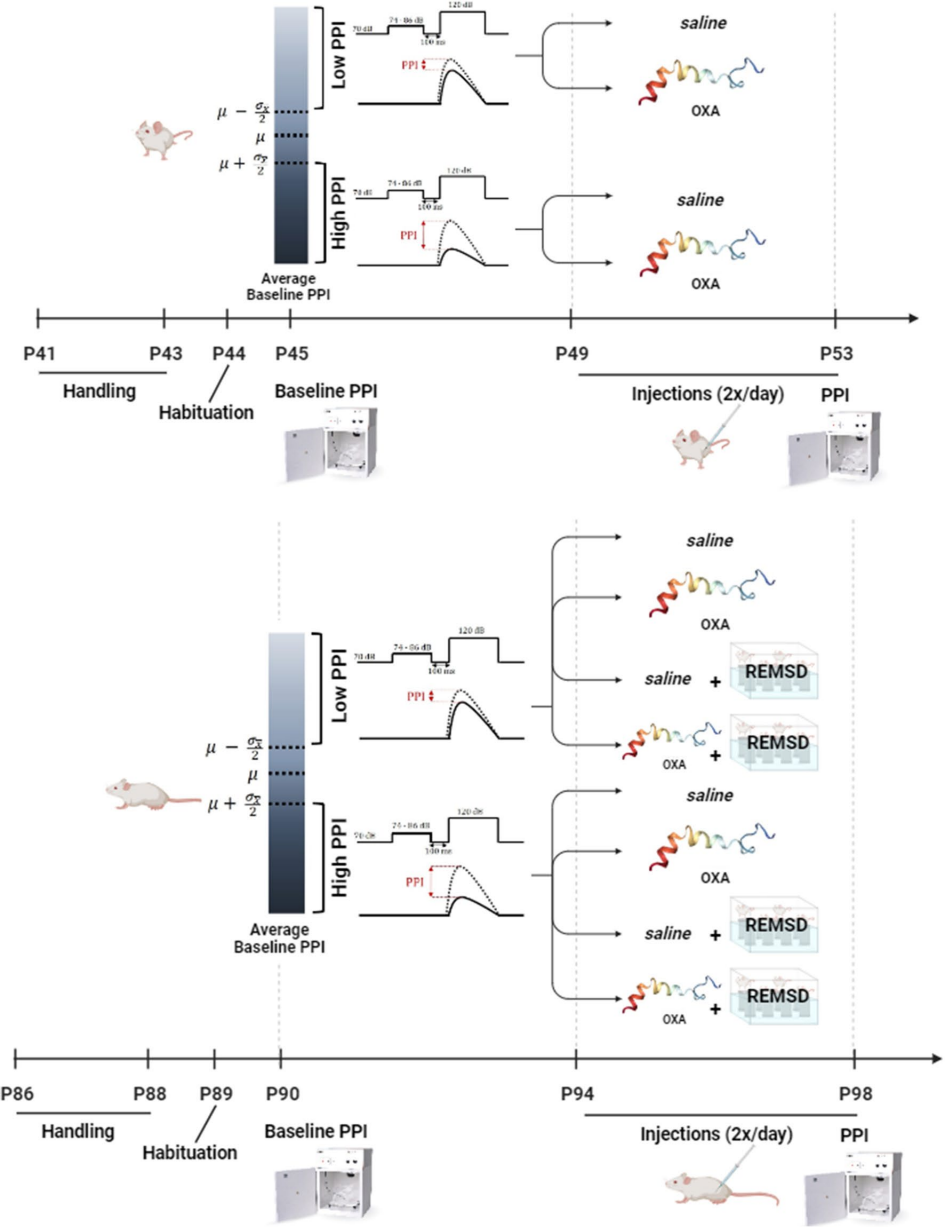
Experimental timeline. The subjects were grouped as low-PPI and high-PPI after baseline PPI measurements on P45 and P90. The PPI protocol included a background noise with 70 dB average intensity, 120 dB pulse intensity and 74,76 or 86 dB prepulse intensities. Prepulses preceeded the pulse by 100 ms. After grouping, OXA injections (2x/day for 4 days) were performed on P49 or on P94. 72 h REMSD and REMSD + OXA applications were performed on P94. On P53 and P98 PPI responses were measured 30 min. after the last injection

The rats were distributed to the groups depending on their average baseline PPI% (bPPI) on day 0 (Fig. 1), such that the group averages ($\overline{\mu })$ over the individual average PPI% of each rat (*µ*) were similar on all groups. The groups were further divided into low-PPI ($\mu <(\overline{\mu }- \frac{\sigma }{2})$) and high-PPI ( $\mu >\left(\overline{\mu }+ \frac{\sigma }{2}\right)$) groups, where $\sigma$ is the standard deviation of the group bPPI. The rats within the band of average bPPI ( $(\overline{\mu }- \frac{\sigma }{2})<\mu <(\overline{\mu }+ \frac{\sigma }{2})$) were omitted (*n* = 6 for P45 and *n* = 9 for P90). The bPPI measurements were taken on either on postnatal day 45 (P45) or P90, and the animals were grouped into either low-PPI or high-PPI groups. Baseline startle for low-PPI and high-PPI rats did not differ significantly.

### Age- and baseline-PPI-dependency of orexin A response

Age-dependency of response to chronic OXA treatment was studied on NSD rats. Rats on P45 and P90 were distributed in one control (-/- as non-REMSD/no OXA) and three treatment (-/+) groups per age, further divided in two subgroups as low-PPI and high-PPI. Four days after bPPI grouping, the treatment groups received two chronic intraperitoneal (*ip*) injections of 2.5 µg/kg, 5 µg/kg or 10 µg/kg OXA (Cayman Chemical, USA) per day for four consecutive days and control groups received only saline of same volume with the treatment group twice per day for the same duration (Fig. 1). All injections were applied 3 h after the start and 3 h before the end of the light period to simulate the abnormal increase in OXA levels during the inactive period of nocturnal rats.

### Baseline-PPI-dependency of REM sleep deprivation

The baseline-PPI-dependency of REMSD was studied on P94 rats (Fig. 1). The baseline PPI grouping was performed as described previously on P90. Animals were subjected to72 hour REMSD in two groups as low-PPI and high-PPI. Modified multiple platform technique was used for 72 h REMSD model (Öz et al. 2018). Four days after bPPI grouping, the rats were placed in a 145 cm × 44 cm × 45 cm transparent plexiglass tank with 14 plexiglass platforms. The tank was filled with water at room temperature to a level 2 cm below the surface of the platforms and covered with grids containing sufficient food and water. A single session lasted for 72 h, with 8–10 rats in the tank per session. PPI% was measured immediately after REMSD. The control group of the age-dependency experiments were used as the control group of REMSD (-/-). The REMSD control group (+/-) also received saline injections twice per day as described for the control group.

### Overlap of REM sleep deprivation and orexin A

Our previous findings indicated that (1) REMSD disturbs PPI% response and (2) acute 10 µg/kg OXA injection after REMSD restores impaired PPI% (Öz et al. 2018). Therefore, the impact of chronic OXA treatment during REMSD was also studied on P98 REMSD rats (Fig. 1). Rats were distributed in three treatment groups (+/+), further divided in two subgroups as low-PPI and high-PPI. The bPPI grouping was performed as described previously on P90. Four days after bPPI grouping, the animals were subjected to 72 h REMSD and received two chronic *ip* injections of 2.5 µg/kg, 5 µg/kg or 10 µg/kg OXA (Cayman Chemical, USA) per day. The injections started 24 h before placing the rats in the REMSD tank. The last injection was delivered 30 min. before removal from the tank. PPI% was measured immediately after the removal from REMSD tank.

### Statistical analysis

Statistical analysis were performed on Matlab R2016a (Mathworks,USA). The age- and bPPI-dependency of OXA treatment was analyzed over high-PPI and low-PPI groups for both age groups. PPI% results were initially analyzed by repeated measures ANOVA with Greenhouse-Geissner correction for PI-treatment interactions. The design included PPI% for three PIs as within subjects and treatment groups as between factors. When PI-treatment interaction was not significant, the groups were compared by one-way ANOVA for each PI and seperately for high-PPI and low-PPI groups. P90 groups share their control (-/-), therefore, only OXA treatment groups (-/ +), only REMSD group(+/-) and REMSD + OXA treatment groups (+ / +) were analyzed together with one-way ANOVA. Startle was also analyzed by one-way ANOVA as described above. Multiple pairwise comparisons were performed by post-hoc Tukey–Kramer test. The results were presented as group averages and standard errors in the tables and figures.

## Results

### Age-dependency of OXA-induced PPI response

The age-dependency and bPPI-dependency of OXA treatment was tested on P53 and P98 (Table 1) over low-PPI and high-PPI rats.

**Table 1:** The summary of prepulse inhibition and startle results

Age	Group	bPPI	n	74 dB	78 dB	86 dB	Startle
P53	Control(-/-)	Low	8	2. 9 ± 1.9	19.2 ± 3.2	40.9 ± 1.9	46.9 ± 10.3
		High	10	22.8 ± 4.7	49.1 ± 3.2	60.2 ± 2.7	41 ± 11.4
	OXA 2.5 µg/kg (-/ +)	Low	8	***34.4* ± *3.8***	***42* ± *4.1***	54.6 ± 5.4	36.6 ± 6.1
		High	8	***52.1* ± *6.3***	64.4 ± 6	68.9 ± 4.9	33.6 ± 7.1
	OXA 5 µg/kg (-/ +)	Low	6	***24.8* ± *6.2***	31.9 ± 7	47.3 ± 9.1	48.9 ± 10.4
		High	6	19.8 ± 11.7	23.4 ± 11	***29.2* ± *12.5***	33.9 ± 10.6
	OXA 10 µg/kg (-/ +)	Low	6	***19.4* ± *4.8***	31.7 ± 4.7	35.4 ± 7.2	27 ± 9.8
		High	7	16.3 ± 6.3	***19.4* ± *9.9***	***21.2* ± *9.6***	13.6 ± 4.3
P98	Control (-/-)	Low	16	31.7 ± 4.4	56.7 ± 3.3	68.3 ± 3.6	101.5 ± 22.8
		High	14	69.1 ± 2.7	80.9 ± 1.2	87.1 ± 1.1	208.9 ± 52.6
	OXA 2.5 µg/kg (-/ +)	Low	6	38.8 ± 10.1	50.8 ± 8.5	83 ± 2.9	170 ± 53.4
		High	7	46.9 ± 10.9	66.5 ± 9.5	86.7 ± 2.9	278 ± 106.1
	OXA 5 µg/kg (-/ +)	Low	6	36.6 ± 8.5	67 ± 4.4	77.6 ± 4.8	185.7 ± 53.3
		High	7	61.1 ± 7.1	73.9 ± 6.9	87.3 ± 2.2	319.5 ± 69.7
	OXA 10 µg/kg (-/ +)	Low	6	39.2 ± 9.1	60.5 ± 7	77.9 ± 4	237.9 ± 100.5
		High	6	47.6 ± 7.4	62.4 ± 5.7	74.9 ± 5.2	87.8 ± 39.4
	REMSD ( ±)	Low	7	48.4 ± 6.7	57.9 ± 5.2	65.5 ± 4.6	67 ± 12.8
		High	6	***27.6* ± *11.3***	***44.4* ± *9.2***	***49.8* ± *14.4***	48.8 ± 13.9
	REMSD + OXA 2.5 µg/kg (+ / +)	Low	6	28.8 ± 12.4	31.3 ± 13.9	42.4 ± 16.3	31.8 ± 10.4
		High	6	***6.7* ± *4.5***	***29.8* ± *7.6***	***37.6* ± *12.6***	16.5 ± 5.4
	REMSD + OXA 5 µg/kg (+ / +)	Low	6	39.9 ± 10.4	45.7 ± 9.2	68.5 ± 5.5	55.1 ± 14.2
		High	6	***20.2* ± *7.7***	***39.8* ± *5.8***	72.2 ± 4.5	94.2 ± 20.4
	REMSD + OXA 10 µg/kg (+ / +)	Low	6	23.1 ± 7.2	34.3 ± 9.1	57.3 ± 11.6	31.1 ± 6.7
		High	7	***22.2* ± *8.4***	***26.3* ± *5.52***	***39.4* ± *10.6***	33.7 ± 17.9

PPI% values are given for each prepulse intensity. All results are given as group averages ± standard error

**Fig. 2:**
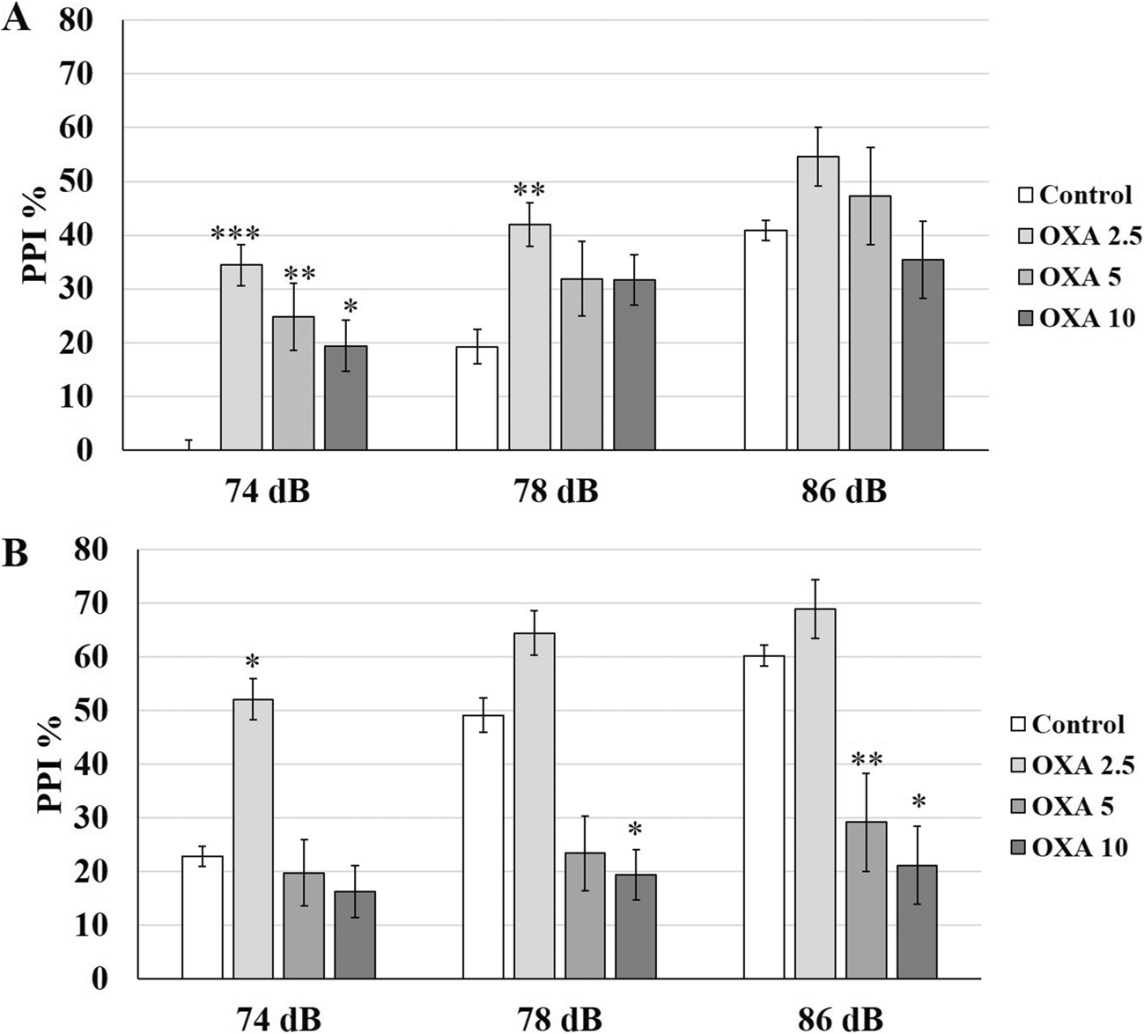
bPPI-dependency of OXA injection on P53 rats. **A** The low-PPI groups displayed elevated PPI% in response to 74 dB and 78 dB prepulse intensities after OXA injections. **B** The high-PPI groups displayed elevated PPI% in response to 2.5 µg/kg OXA, however, the response was attenuated for higher doses. * *p* < 0.05, ** *p* < 0.005,*** *p* < 0.0005. The error bars represent standard errors

**On P53,** no interaction was detected between PI-treatment in either high-PPI (*p* = 0.35) or low-PPI (*p* = 0.25) groups. For low-PPI groups, PPI% was elevated for 74 dB PI compared to control (-/-) group by 2.5 µg/kg (*p* = 0.0001), 5 µg/kg (*p* = 0.005) and 10 µg/kg OXA (*p* = 0.04) (Fig. 2A). The increase for 2.5 µg/kg was the highest with 31% and decreased gradually to 21.9% and 16.5% for 5 µg/kg and 10 µg/kg (Table 1). 2.5 µg/kg OXA also significantly elevated PPI% for 78 dB PI (*p* = 0.008). The effect of OXA was opposite for high-PPI rats (Fig. 2B). PPI% for 86 dB PI was significantly attenuated by 5 µg/kg (*p* = 0.002) and 10 µg/kg OXA (*p* = 0.03) compared to control (-/-) group. Only 10 µg/kg OXA significantly reduced PPI% for 78 dB PI (*p* = 0.02). Interestingly, 2.5 µg/kg OXA elevated PPI% for all PIs but the increase was significant only at 74 dB (*p* = 0.02). OXA treatment did not yield any significant effect on startle response in either high-PPI or low-PPI groups.

**Fig. 3:**
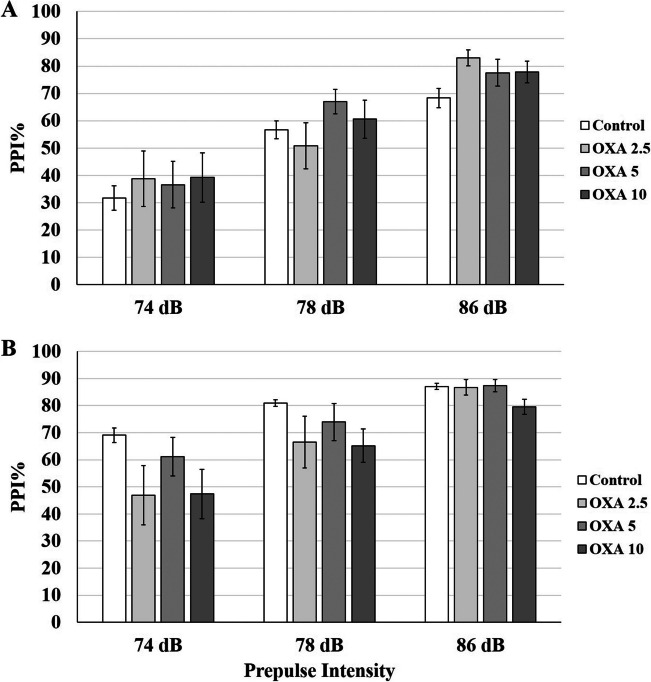
bPPI-dependency of OXA injection on P98 rats. The PPI% response was not altered after OXA injection neither in low-PPI (**A**) nor in high-PPI (**B**) groups. * *p* < 0.05, ** *p* < 0.005,*** *p* < 0.0005. The error bars represent standard errors

**On P98,** the PI-treatment interaction was not significant in either high-PPI (*p* = 0.72) or low-PPI (*p* = 0.32) groups, therefore, each treatment groups were distributed over PIs and startle. None of the OXA doses had a significant effect on PPI% for 74 dB, 78 dB and 86 dB PIs and on startle in neither low-PPI (Fig. 3A) nor high-PPI groups (Fig. 3B).

These results imply that the effect of OXA on PPI% is bPPI- and age-dependent. In younger rats, the effect is improving for low-PPI rats and disruptive at higher doses for high-PPI rats.

### The effect of REMSD on PPI

The effect of 72 h REMSD alone (+/-) was compared to control (-/-) group in high-PPI and low-PPI animals to reveal the bPPI-dependency of the efficiency in REMSD models, and to OXA treatment groups to understand how OXA stimulation impact REMSD-induced PPI% impairment in a bPPI-dependent manner. There was no significant interaction between PI-treatment in either high-PPI (*p* = 0.07) or low-PPI (*p* = 0.06) groups.

**Fig. 4:**
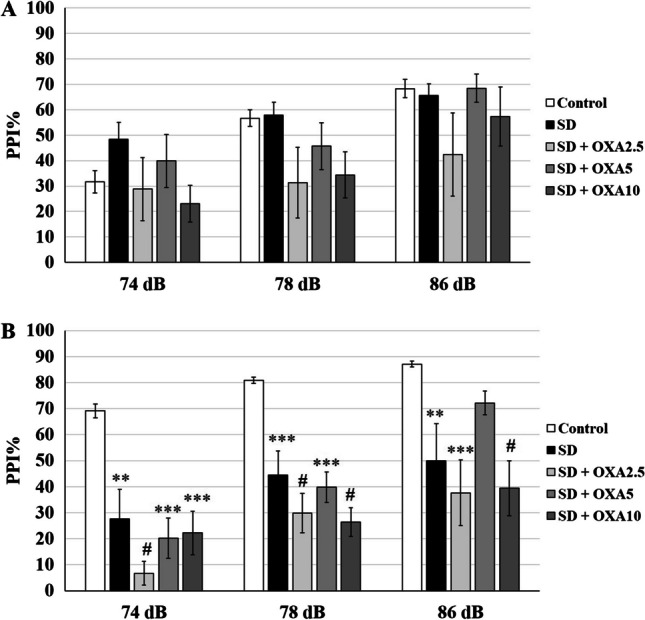
bPPI-dependency of REMSD on P98 rats. **A** 72 h REMSD did not significantly alter PPI% response in low-PPI animals. **B** REMSD significantly attenuated PPI% in high-PPI animals for 74 dB and 78 dB prepulse intensities. * *p* < 0.05, ** *p* < 0.005,*** *p* < 0.0005. The error bars represent standard errors

When compared to control (-/-) group, 72 h REMSD differentially affected low- and high-PPI rats. REMSD model was not effective on low-PPI group at any PI (Fig. 4A). On the contrary, REMSD attenuated PPI% in high-PPI rats at 74 dB (*p* = 0.0016), 78 dB (*p* = 0.0007) and 86 dB PIs (*p* = 0.0001) (Fig. 4B). Startle was not affected by REMSD in either high-PPI or low-PPI groups.

**Fig. 5:**
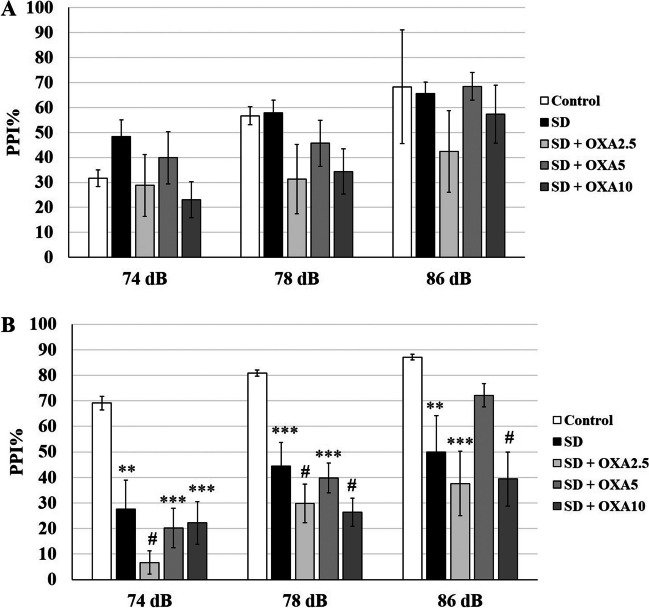
The combined effect of REMSD and OXA on P98 rats. **A** Combined 72 h REMSD and OXA injections did not alter PPI% in low-PPI animals. **B** The PPI% was significantly attenuated in high-PPI animals, however, 5 µg/kg OXA restored PPI% impairment only for 86 dB prepulse intensity. * *p* < 0.05, ** *p* < 0.005,*** *p* < 0.0005. The error bars represent standard errors

**Fig. 6:**
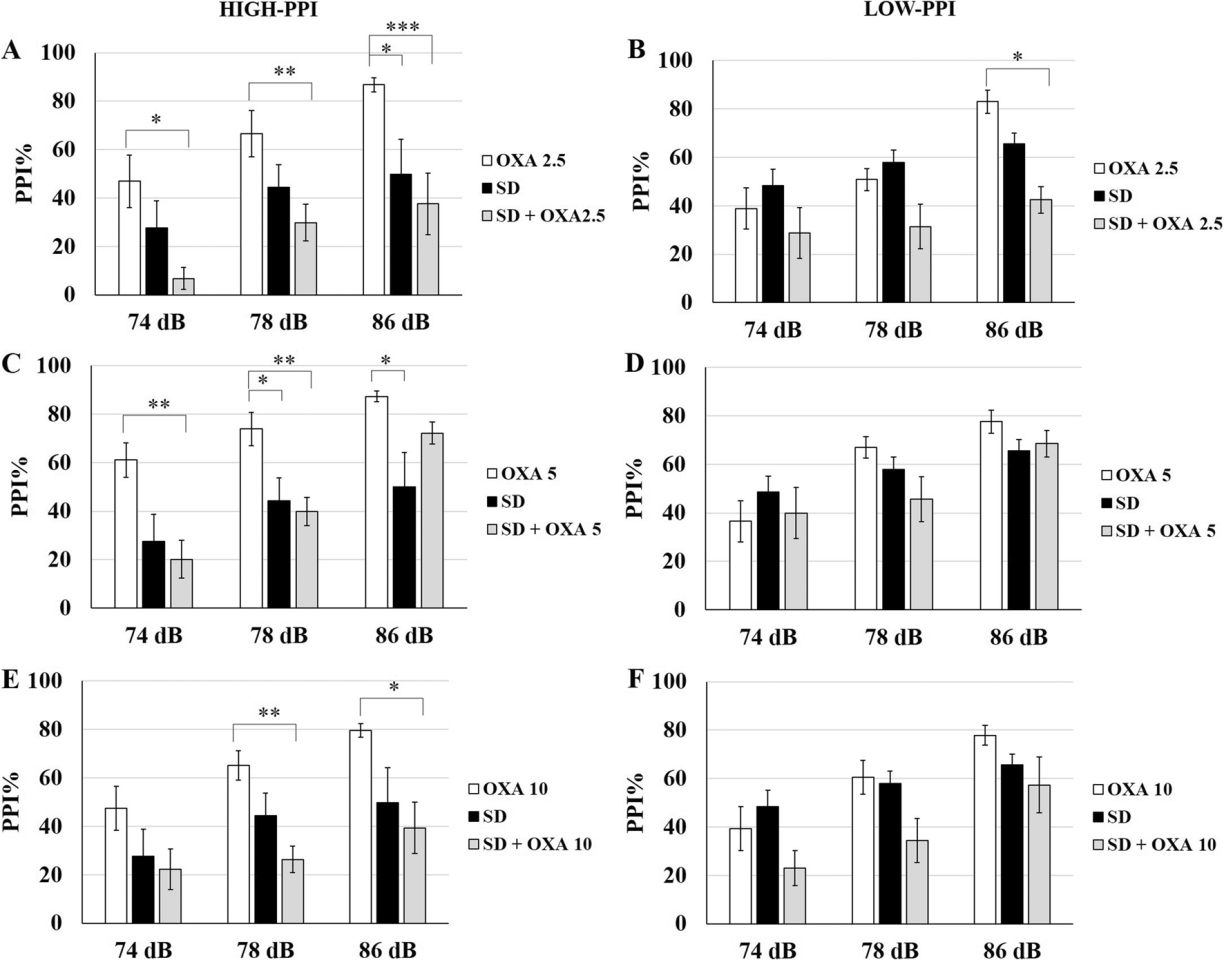
The comparison of OXA, REMSD and REMSD + OXA on P98 rats. The impact of the REMSD + OXA combination was compared to the OXA injections alone and control groups for high-PPI (**A, C, E**) and low-PPI (**B, D, F**) animals. * *p* < 0.05, ** *p* < 0.005,*** *p* < 0.0005. The error bars represent standard errors

The response to chronic OXA treatment together with REMSD also displayed a dependence on bPPI levels. In low-PPI rats, OXA treatments with REMSD did not create a significant effect at any PI compared to control (-/-) or REMSD ( ±) groups (Figs. 5A and 6B, D, F). In high-PPI rats, none of the REMSD + OXA groups had any significant difference in PPI% from REMSD group for any of the PIs (Figs. 5B and 6A, C, E). PPI% was still significantly lower than control (-/-) group for 2.5 µg/kg OXA for 74 (*p* < 0.0001), 78 dB (*p* < 0.0001) and 86 dB PIs (*p* = 0.0001). Similarly, 10 µg/kg OXA with REMSD resulted in lower PPI% compared to control (-/-) group for 74 (*p* = 0.0001), 78 dB (*p* < 0.0001) and 86 dB PIs (*p* < 0.0001). 5 µg/kg OXA with REMSD also resulted in lower PPI% compared to control (-/-) group for 74 (*p* = 0.0001), and 78 dB (*p* = 0.0001), however, it restored the REMSD-induced PPI% reduction for 86 dB. REMSD and OXA combination did not have any effect on startle in either low-PPI or high-PPI groups.

## Discussion

Even though OXA levels are associated with the severity of symptoms in schizophrenia (Chien et al. 2015; Liu et al. 2020; Ni et al. 2019), where PPI impairment is a common diagnostic marker (Mena et al. 2016), the information on the direct relationship between OXA and PPI is limited (Abbas et al. 2015; Öz et al. 2018, Demidova et al. 2022). Previously, we studied this relationship with acute OXA treatment on both NSD and sleep-deprived rats (Öz et al. 2018) and showed that high acute doses negatively affect PPI for NSD rats. Currently, we show that the chronic effect of OXA depends on bPPI levels and age.

### OXA-induced modulation of PPI response is age-dependent

OXA-induced modulation of PPI response was only observed at P53, which suggests an underlying mechanism for the OXA responsiveness that is being altered by age. This finding implies that a dynamic interplay between age-related factors and OXA may alter the brain processes responsible for modulating PPI responsiveness. Previous investigations have revealed that orexin function, either at the expression or receptor level, declines with age (Matsumura et al. 2002; Terao et al. 2002; Porkka-Heiskanen et al. 2004; Kessler et al. 2011). This is consistent with the literature and shows a clear impairment of OXA-induced modulation of the PPI response at P98. Our findings also agree with previous reports indicating that PPI response is elevated by age (Jafari et al. 2020; Choy et al. 2021). Together with the age-related changes in orexinergic system, the developmental changes on the PPI-related systems could also be one of the underlying mechanisms. Understanding the complex mechanisms underlying OXA-induced age-dependent modulation of PPI responsiveness may shed light on a variety of neurological processes.

### OXA-induced modulation of PPI response is bPPI-dependent

In laboratory rodent populations, bPPI displays a natural spectrum. The low end of this spectrum is suggested to reflect an inherent tendency for psychosis-like condition and dopamine sensitivity (Peleg-Raibstein et al. 2013). Since higher OXA levels were associated with less severe symptoms in schizophrenia (Chien et al. 2015; Liu et al. 2020), our expectation was to observe improved PPI% in low-PPI rats after OXA treatment. This expectation was confirmed by our findings, however, only for P53 animals. In low-PPI rats, OXA elevated PPI response especially at lower PIs. At the lowest PI, which is only 4 dB higher than the background noise, 2.5 µg/kg chronic OXA injection was enough to increase the PPI% and even though the effect persisted for higher doses as well, the effect at the lowest dose was the highest. This implies that lower doses of OXA might be beneficial for models that can be associated with low-PPI and that the mechanism behind the innate low-PPI response might involve impaired orexinergic activity. We therefore propose that low-PPI animals can be considered as a model to study sleep-related disorders in neurodegenerative and neurodevelopmental disorders, for which PPI impairment is a diagnostic marker.

Chronic OXA injection during day, which is the sleep period for nocturnal animals, was expected to induce abnormal wakefulness-like activity in neurochemical systems and therefore impair sensorimotor gating in animals that do not have a preexisting PPI impairment. This effect was only observed for high-PPI rats, which are considered as the “healthy” group, on P53.

In our previous study with acute OXA treatment (Öz et al. 2018), the groups were not classified over bPPI levels, instead, both low-PPI and high-PPI rats were distributed into control and experimental groups with equal weight. The negative effect of high doses of OXA on these groups agree with our findings on high-PPI rats and it might be explained with the excess stimulation by OXA. It should also be noted that acute treatment in our previous study was applied after REMSD to test a reversal effect. However, OXA treatment was initiated before REMSD and was continued throughout in this study, to test possible protective effects.

OXA injection during day did not affect startle levels neither on P53 nor P98, which suggests the age- and bPPI-dependent impact of OXA on PPI% was achieved via PPI mediation and/or modulation networks.

### REMSD acts differentially on PPI in low-PPI and high-PPI rats

Previous studies reported successful therapeutical use of SD against bipolar disorder and major depression (Benedetti and Colombo 2011; Sikkens et al. 2019; Ramirez-Mahaluf et al. 2020). These studies already point towards a diverse effect of SD depending on the inherent disorders, which was reflected by bPPI in our study. Our results indicate that the effect of 72 h REMSD on PPI% is bPPI-dependent. In low-PPI group, REMSD does not further attenuate the PPI%. It was previously shown that REMSD increases OXA levels (Mehta et al. 2015), however, our previous results already suggest decreased OXA responsiveness in low-PPI animals. Therefore, decreased OXA responsiveness could still be an explanation for our findings. Another explanation could be that, in low-PPI animals, the sensorimotor gating system is already not functioning and any other factor is, therefore, ineffective. This explanation is, however, contradicting with the OXA-induced elevation of PPI response for low-PPI animals on P53. Opposite to low-PPI rats, PPI% impairment in high-PPI rats demonstrate that the REMSD model of psychosis is only effective when the rat does not have low bPPI. REMSD did not have an effect on startle response in both high-PPI and low-PPI animals, which was consistent with the literature (Kaya Yertutanol et al. 2020). Therefore, similar to OXA injections, it can be proposed that REMSD-induced PPI% impairment was due to altered activity in PPI mediation and/or modulation networks.

Although REMSD is considered as a valid approach for modelling psychosis-like symptoms in rodents, one criticism would be that its effect on both psychopathology and biomarkers of schizophrenia remains limited (Kumari and Ettinger 2020). Therefore, a combination of SD with other models of psychosis was previously suggested. Hereby, we propose that the analysis of REMSD on a group of rodents with varying bPPI already provides such a combination, i.e. low-PPI can be readily considered as a naturally occurring model (Peleg-Raibstein et al. 2013). However, this combination interestingly does not have a compound effect on PPI.

We would also like to emphasize that, bPPI might inadvertently affect the success of the REMSD models and the outcome, i.e. in symptomatic animal models of schizophrenia, and therefore, we recommend either grouping by bPPI levels or working only with high-PPI animals in such studies.

### REMSD and Orexin

Key parameters on the relationship of SD and orexinergic system seems to be the type and duration of SD. Previous studies utilized various durations of either total SD, REMSD or sleep fragmentation to understand the role of orexinergic system (Deadwyler et al. 2007; Arthaud et al. 2015; Matsuki et al. 2015; Mehta et al. 2015; Ran et al. 2015; Toossi et al. 2016; Briggs et al. 2018, 2019). Plasma OXA level was shown to decrease in rats following 24 h REMSD (Ran et al. 2015) and to increase in insomnia patients (Tang et al. 2017). Orexin levels in cerebrospinal fluid were also higher in Alzheimer’s Disease patients with mild cognitive deficits, which was also correlated with prolonged sleep latency, reduced sleep efficiency and REM sleep impairments (Liguori 2016). These findings suggest that the activity of the orexinergic system may depend on the type and duration of sleep disturbances.

Previous studies suggest that the diverse findings from different sleep disturbance cases might be due to the modulation of orexinergic activity at the synapses, rather than the regulation on gene expression or other intracellular events. In the case of 6 h total SD, the activity of D-type orexin neurons and the presynaptic inhibition of sparse excitatory inputs in both D-type and H-type orexin neurons increased (Briggs et al. 2019), while it also reduced the astrocytic modulation of glutamatergic synapses around orexinergic neurons via reduced glutamate uptake (Brigss et al. 2018). SD and orexinergic system activity is closely related with the GABAergic activity on orexinergic neurons and also with the innervation of GABAergic neurons by orexinergic neurons. The number of GABA-A-receptor-positive orexinergic neurons and of GABA-A receptors on these neurons were increased after SD (Toossi et al. 2016). Both acute and chronic SD reduced efficacy of OXA in VLPO, although more strongly after chronic REMSD (de Porter et al. 2017). This reduction in efficacy also seems to be increased after a prolonged duration of sleep loss (de Porter et al. 2017; Carter et al. 2009). While these findings point to synaptic regulation after SD, the expression of preproorexin in hypothalamus was found to be independent from the duration of SD (Martins et al. 2010), confirming the idea that the regulation is probably not via intracellular mechanisms.

Acute low dose treatment of OXA after 72 h REMSD was previously shown to restore impaired PPI, specifically at 78 dB PI, where the most severe impairment was observed (Öz et al. 2018). This finding was in agreement with the idea that lower OXA levels after REMSD (Ran et al. 2015) might be a factor in PPI impairment. Our results show that, when administered chronically, OXA does not provide a similar improvement as for acute treatment. Even more interestingly, the impact of orexinergic activation was dependent on bPPI levels. For low-PPI rats, REMSD already did not have an impact on PPI%, and OXA treatment did not alter this. However, OXA treatment also did not have an effect on the attenuated startle response after REMSD. For high-PPI rats, neither impaired PPI% could be fully restored by chronic OXA treatment (aside the singular improvement by 5 µg/kg OXA for 86 dB PI) nor PPI% was impaired more severely. With the exception of 5 µg/kg OXA, the treatment also did not affect the attenuated startle response after REMSD. An explanation could be that the impairment after REMSD could not be restored due to the involvement other neurotransmitter systems, which are independent of OXA, and their altered activity could not be recovered by repeated OXA treatment. Together with the previous findings of glutamatergic and GABAergic regulation of orexinergic activity after REMSD (Toossi et al. 2016; Briggs et al. 2018, 2019), we believe it is crucial to uncover the exact mechanism behind the modulation of orexinergic system after REMSD and it could be expected that the key elements of this regulation is also involved in PPI mediation/modulation. As an alternative explanation, prolonged duration of OXA treatment during REMSD may not be as effective as an application immediately after. The questions on the dependence of REMSD effect on bPPI and the involvement of orexinergic system in SD-related networks, together with the mechanism that reflects the PPI impairment, demand a closer look on the regulation of neural activity in PPI-related networks.

## Conclusion

Our findings imply two important conclusions: First, the success of 72 h REMSD model depends on bPPI and the model is ineffective on low-PPI rats. Second, even though orexinergic activity clearly affects PPI, its impact on PPI after REMSD is limited. Both of these findings demand detailed investigation, i.e. on the role of orexinergic system in PPI-related networks.

### Supplementary Information

Below is the link to the electronic supplementary material.

Supplementary file1 (PDF 258 KB)
