# Supplementary material for: Baseline prepulse inhibition dependency of orexin A and REM sleep deprivation
Source: Psychopharmacology (Berl). 2024 Mar 1;241(6):1213–25. doi: 10.1007/s00213-024-06555-3 (PMC11106105; doi:10.1007/s00213-024-06555-3)
Supplement: Supplementary file 1 — Supplementary file1 (PDF 258 KB) [file 213_2024_6555_MOESM1_ESM.pdf]

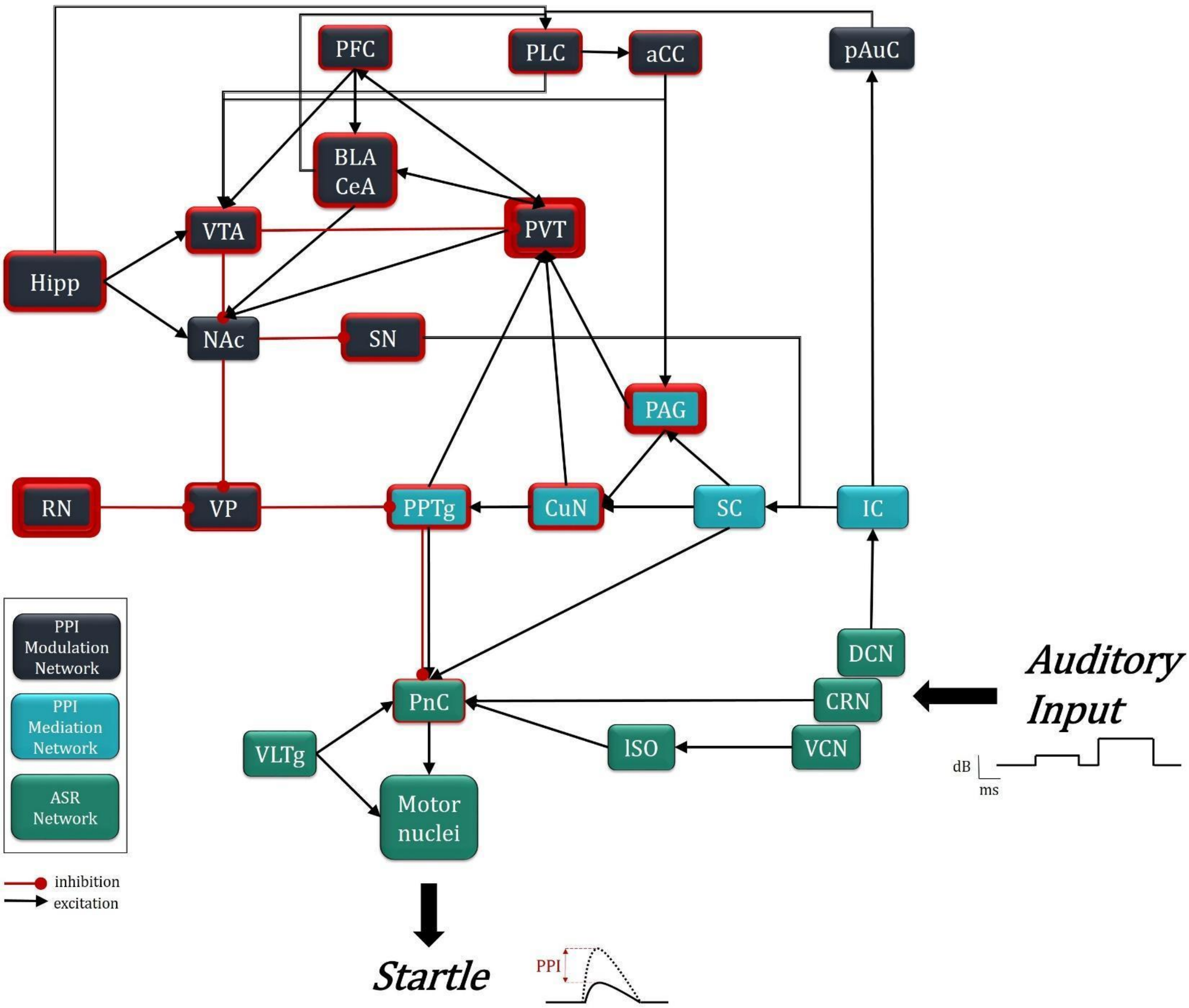

Supplementary Figure 1: The proposed network for prepulse inhibition of acoustic startle reflex. The network proposed in Öz et al. (2018) is expanded to include more targets for orexinergic innervation. The red borders represent orexinergic inputs, scaled with relative densities **Abbreviations :** aCC : anterior cingulate cortex, BLA : basolateral amygdala, CeA : central amygdala, CRN : cochlear root neurons, CuN : cuneiform nucleus, DCN : dorsal cochlear nucleus, Hipp : hippocampus, IC: inferior colliculus, NAc : nucleus accumbens, PAG : periaquadectal gray, pAuC: primary auditory cortex, PFC: prefrontal cortex, PLC : prelimbic cortex, PnC : pontine reticular formation, PPTg : pedunculopontine tegmental nucleus, PVT: paraventricular thalamic nucleus, RN : raphe nuclei, SC: superior colliculus, SN : substantia nigra, VCN : ventral cochlear nucleus, VLTg : ventrolateral tegmental nucleus, VP : ventral pallidum, VTA: ventral tegmental area.
